# Improved performance of Xpert MTB/RIF assay on sputum sediment samples obtained from presumptive pulmonary tuberculosis cases at Kibong’oto infectious diseases hospital in Tanzania

**DOI:** 10.1186/s12879-017-2931-6

**Published:** 2017-12-29

**Authors:** Peter M. Mbelele, Said Aboud, Stellah G. Mpagama, Mecky I. Matee

**Affiliations:** 1 Kibong’oto Infectious Diseases Hospital, P.O BOX 12 Sanya Juu, Kilimanjaro Tanzania; 2 Department of Microbiology and Immunology, School of Medicine, Muhimbili University of Health and Allied Sciences, P.O BOX 65001 Dar es Salaam, Tanzania

**Keywords:** Tuberculosis, Xpert® MTB/RIF, Sputum-sediments, Presumptive-tuberculosis, Kibong’oto, Tanzania

## Abstract

**Background:**

The introduction of Xpert MTB/RIF assay (Xpert) has significantly improved diagnosis of Tuberculosis (TB) in resource limited human immunodeficiency virus (HIV) endemic settings. We aimed to modify the Xpert protocol to improve the detection of *Mycobacterium tuberculosis* (MTB).

**Methods:**

This cross sectional study was conducted among presumptive pulmonary tuberculosis (PTB) patients at Kibong’oto Infectious Diseases Hospital between August and November 2015. Each patient consented to provide 2 samples of raw sputa. One-sputum sample was sedimented using the Petroff’s method and divided into two portions. One portion of sediment was inoculated on Lowenstein-Jensen culture media and observed for any growth for up to 8 weeks. Both, raw sputum and the portions of sediments were tested separately using Xpert with a sample reagent ratio of 1:2. Mean age of patients, prevalence of MTB, Xpert sensitivity, specificity, positive and negative predictive value were calculated. An incremental sensitivity was determined. Pearson chi-square and either an independent T or Mann-Whitney U-test were used to compared categorical and continuous variables respectively. A p- value of ≤0.05 was considered significant.

**Results:**

Of the 270 presumptive PTB cases, 262 were eligible for analysis. Eight (3%) were excluded due to contaminated culture. Patients’ mean age was 42.9 (±SD 15.1) years of which 173 (66%) were female. The overall prevalence of PTB was 112 (43%), of which the Xpert detected 105 (40%) in sediments and 98 (37%) in raw sputa as compared to culture which detected 85 (32%) cases of PTB. Sensitivity, specificity, positive and negative predictive values of Xpert on sputum sediments were 92%, 85%, 74% and 96% respectively. Overall, the incremental sensitivity of Xpert on sediment over raw sputum was 6%. In HIV infected Presumptive PTB, the incremental sensitivity was 12%.

**Conclusion:**

Lowering the sample reagent to sediment dilution ratio increases sensitivity of Xpert on MTB detection among presumptive PTB cases, especially in HIV infected individuals.

## Background

Tanzania remains one of the countries with a high burden of tuberculosis (TB) and human immunodeficiency virus (HIV) co-infection [1]. Despite the consolidated control strategies, the incidence of TB and mortality rate in both TB and TB with HIV co-infected cases has remained steady [2]. A key challenge in TB/HIV control includes the difficulty in correct diagnosis due to alteration of clinical presentation resulting in a delay of appropriate treatment [3]. Important factors that delay early diagnosis of TB include the inability of TB/HIV co-infected patients to produce quality sputum for diagnosis [4]. Previously studied methods for improving the quality of sputum for TB diagnosis include overnight pooling [5] and processing sputum with different chemicals such as N-Acetyl-L-Cysteine (NALC) to concentrate *Mycobacterium tuberculosis* (MTB) [6]. Recent technologies to increase the sensitivity of MTB detection in samples include light emitted diode microscopy [7], and rapid molecular diagnostics such as Xpert [8].

The introduction of Xpert (Cepheid, Sunnyvale USA) in particular has been a major breakthrough in TB diagnostics, especially in resource limited HIV endemic settings. The assay is a semi-automated real time Polymerase Chain Reaction (PCR) that was approved by the World Health Organization (WHO) in 2010 for dual detection of MTB and rifampicin resistance [8]. A systematic review of Xpert has shown an excellent performance compared to conventional smear microscopy for acid fast bacilli (AFB) and Liquid or Lowenstein Jensen (LJ) solid culture methods. Compared with smear microscopy, a point of care test for TB diagnosis in most resource limited settings, Xpert considerably increases TB detection among culture-confirmed cases accounting for pooled sensitivity and specificity of 89% and 99% respectively [9]. However, Xpert has higher sensitivity in detecting MTB in samples that were collected from patients with smear-positive results for AFB than smear-negative [10]. Smear negativity is a common phenomenon among HIV positive patients and the performance of Xpert on pooled samples of HIV individuals was 79% and 86% sensitivity and specificity respectively [9]. However, like other molecular diagnostics, Xpert suffers a diminished effect in the HIV population [11].

The commonly applied protocol for detecting MTB in Xpert at our hospital and in most other settings is the use of raw sputum to Xpert sample reagent (SR) with a ratio of 1:2 [10, 12]. The SR contains 2% of sodium hydroxide (NaoH) and isopropanol. However, manufacturer of Xpert recommends the use of either raw, unprocessed sputa or concentrated sputum sediments. Here, the raw sputum sample is liquefied, decontaminated either with 2% N-acetyl cysteine-sodium hydroxide (NALC-NaOH) or 4% NaoH, centrifuged, concentrated and neutralized using phosphate buffer [13]. These sediments have higher load of MTB compared to raw sputum [14]. Nevertheless, previous reports for MTB detection in Xpert using processed sputum sediments did not show any difference to raw sputum [10, 14]. These studies reportedly used a raw and processed sputum to SR ratio of 1:2 and 1:3 respectively [15]. Using sputum sediments from patients with TB/HIV co-infection, Dharan et al. [16] found high detection rate of MTB when the sediment/SR ratio was changed to 1:2. Our hypothesis is that decreasing the Xpert SR dilution will increase the probability of detecting MTB among PTB suspects in resource limited HIV endemic settings like ours. While the raw sputum was processed as per manufacturer’s protocol, we sought to modify the Xpert protocol by particularly adjusting the dilution of sediment to SR with a ratio of 1:2 instead of 1:3 as recommended. Using LJ culture medium as a reference method, we determined the incremental detection value of MTB on sputum sediment samples.

## Methods

### Study design and setting

This cross sectional study design was conducted at Kibong’oto Infectious Diseases Hospital (KIDH) located in Siha District, Kilimanjaro region, Tanzania. KIDH is a public hospital with bed capacity of 320. It is the National Centre of excellence for clinical management of drug resistant TB in the country. The hospital provides TB services to more than 150 and 500 patients with drug resistant and susceptible TB per year respectively. KIDH has a public health laboratory supported by East African Public Health Laboratory Networks through the World Bank Scheme. Since 2016, the laboratory has established drug resistance TB surveillance system and is now in the process of accreditation. The laboratory has facilities for mycobacterial culture on LJ solid media, smear microscopy for AFB (LED microscopy), Xpert (Cepheid, USA) and Line Probe assay (GenoTypeMTBDR_*plus*_ that detects MTB; and isoniazid and rifampicin susceptibility and GenoTypeMTBDR_*sl*_ for detecting MTB and their susceptibility to second line injectable drugs and flouroquinolones). The average number of sputum samples processed per day for culture and molecular testing ranges from 25 to 30 samples.

### Recruitment and evaluation of study participants

Participants were presumptive pulmonary TB cases aged ≥ 18 years and were enrolled after obtaining a written informed consent. A standardized semi-structured questionnaire containing a set of study variables was used to collect data from study participants and medical charts. Data collected included symptoms and signs suggestive of PTB, any previous history of TB treatment, HIV status, absolute CD4 + T cell count and socio-demographic characteristics such as age, gender, occupation etc.

### Sample size calculations

A minimum sample size of 256 was determined using Buderer’s formula for diagnostic tests. A prevalence of TB among TB suspect cases of 33.5% reported by Meremo et al. [17] and anticipated average Xpert sensitivity for raw sputum and sediment of 95% at a significance level of 5% was used [14]. Because of an anticipated culture contamination rate of about 5% on LJ media, 14 participants were added to the sample size. Therefore a total of 270 participants were enrolled.

### Study procedures

#### Samples collection and processing

Each study participant provided two spot sputum samples 30 min apart. One of the two samples was processed using the modified Petroff’s method [18]. Briefly, 3mls of sputum was added to 3mls of 4% sodium hydroxide (NaOH). The mixture was vortexed and left to stand at room temperature for 15 min. Thereafter, sterile distilled water was added to a 50mark of falcon tube and concentrated by centrifugation at 3000 g for 15 min. Supernatants were discarded into a container with 25% phenol. Sediments were suspended in Phosphate buffer solution (PBS) before being split into 2 portions, one for testing with the Xpert and the other for the LJ culture media as recommended by the Clinical and Laboratory Standard Institute (CLSI) for TB culture [13]. The second unprocessed raw sputum was for direct testing using the Xpert.

#### Detection of MTB in raw sputum and sediments collected from presumptive PTB patients by Xpert

The raw sputum was tested as recommended by the manufacturer of Xpert, here referred to as standard dilution. Unprocessed raw sputum was diluted with sample reagent (SR) at a ratio of 1:2 [15]. Sputum sediments were tested using a modified protocol, referred to as experiment dilution. In this protocol, sediments were diluted with SR at a ratio of 1:2. Both, raw and sediment samples were incubated at room temperature for 15 min to reduce MTB viability by 10^6^ fold as recommended by the manufacturer. At least 2 ml of either raw or sediments sample was transferred into the cartridge and loaded into the Xpert module to continue with automatic DNA extraction, amplification and detection of MTB*.* The MTB detection is done by amplifying MTB specific sequence of the *rpoB* gene probed with five molecular beacons A, B, C, D and E, each labeled with a unique fluorophore. During detection, the valid maximum cycle threshold (CT) was 39.0 for Probes A, B and C and 36.0 for Probes D and E. The MTB is detected when at least two probes result in CT values within the valid range and a delta CT min of less than 2.0. Depending on the CT value, the MTB is semi-quantified into very low, low, medium and high for CT values of >28, 23–28, 16–22 and <16 cycles respectively [19]. Similarly, the assay does not detect MTB when there is only one or no positive probe.

#### Culture of MTB on Lowenstein Jensen (LJ) medium

Part of the sputum sediment was cultured on LJ solid medium, a reference method, as per CLSI [13]. In summary, 200 μl of sputum sediments were inoculated on two slopes of LJ medium containing either pyruvate or glycerol. For each batch of the sputum sample cultured, a standard laboratory strain MTB H37Rv strain and un-inoculated LJ medium was used as positive and negative quality control of culture, respectively. Inoculated LJ media were incubated at 37 °C and observed weekly for up to 8 weeks before declared negative. MTB colonies were identified and reported according to locally existing and CLSI standard operating procedures.

#### Data management and statistical analysis

The semi-structured questionnaire was used as a gold standard tool for data collection from participants and medical charts. Raw data collected were verified for correctness before being entered into EpiData software version 3.1. Data on EpiData were cleaned before analysis using Statistical Package for Social Sciences version 23.0. They were summarized in proportion with percentages or estimated with measure of central tendency with standard deviation of the mean or 95% confidence interval accordingly. Sensitivity, specificity, positive and negative predictive values with 95% CI of Xpert on raw sputum and sediments was calculated using diagnostic test evaluation calculator- MedCalc Statistical Software version 16.4.3 (MedCals software bvba, Ostend, Belgium; https://www.medcalc.org; 2016) [20]. Incremental value of Xpert on raw and sputum sediment was also calculated using the LJ culture as a reference method. The Pearson Chi-square was calculated for categorical variables such as gender, occupation, HIV status and presenting symptoms. Independent T-test and Mann–Whitney U test were used to compare PTB cases with continuous variables like age and absolute CD4+ T cell count respectively. A two-tailed test statistic was considered significant for a *p*-value of ≤ 0.05.

## Results

### Characteristics of study participants

**Fig. 1:**
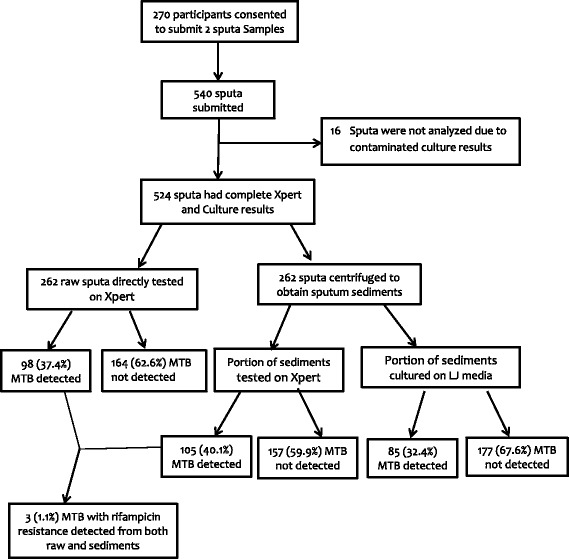
Study Procedures and proportion of PTB

From August through November 2015, 270 presumptive PTB participants consented and provided 2 samples of raw sputa making a total of 540 sputa. However, 16 sputa samples that were collected from 8 participants were not analyzed due to culture contamination (Fig. 1). Therefore, 262 raw sputa were tested on Xpert and the other 262 decontaminated sputa were centrifuged to obtain sputum sediments. Of the 262 participants; 173 (66%) were female. Their mean age was 42.9 (±SD 15.1) years (Table 1). Thirty nine (14.9%) and 36 (13.7%) of them had HIV infection and prior exposure to anti-TB treatment respectively (Table 1). The median absolute CD4 + T cell counts of HIV infected participants was 285 (IQR: 162–423) cells/μL. The main clinical presenting symptoms included cough in 262 cases (100%), hemoptysis in 41 (16%), chest pain/difficulty breathing in 240 (92%) cases, fever in 241 (92%), excessive night sweat in 172 (66%) and weight loss in 203 (78%) cases.

**Table 1:** Baseline characteristics of study participants (*N* = 262)

Characteristics	Frequency	%
Age group (years)		
≤ 24	28	10.7
25–34	46	17.6
35–44	87	33.2
45–54	47	17.9
55+	54	20.6
Sex		
Male	89	34
Female	173	66
Occupation		
Employed	8	3.1
Small business	60	22.9
Peasant	168	64.1
Mining casual workers	26	9.9
HIV status		
Positive	39	14.9
Negative	223	85.1
Median CD4+ T cells counts; n (IQR) cells/μL	285 (162–423)	
History of prior TB treatment		
Yes	36	13.7
No	226	86.3

### Performance characteristics of Xpert and incremental sensitivity on raw and sediment samples

Almost all samples that were collected from study participants had valid Xpert results. These results were similar to those obtained from the standard MTB H37Rv strain and de-ionized water for positive and negative quality control samples respectively. However, four raw sputum samples showed no Xpert results due to prolonged interruption of the laboratory’s power supply. The results were valid when Xpert was repeated. Similarly, one sediment sample had MTB with indeterminate rifampicin resistance (RR). This sample had MTB without RR when Xpert was repeated. The prevalence of PTB detected either by Xpert on raw sputa and sediments or LJ culture method that was collected from presumptive PTB patients was 112 (43%). The Xpert detected 105 (40.1%) on sputum sediments while detection on raw and culture were 98 (37.4%) and 85 (32.4%) respectively (Fig. 1). However, the Xpert detected 3 (1.1%) cases with rifampicin resistance from both raw and sediments (Fig. 1). Of PTB cases detected on sediments, the Xpert semi-quantified MTB as very low 6 (6%), low 27 (26%), moderate 37 (35%) and high 35 (33%).The overall sensitivity of Xpert on sputum sediments was higher than that on raw sputa. Notably, sensitivity in HIV infected presumptive PTB cases was excellent (Table 2). Incremental sensitivity value for all study participants was 6%, while for the HIV infected PTB cases it was 12%. Also, the Xpert detected MTB in 27 (10%) of presumptive PTB cases that were negative on LJ culture, a gold standard. Further examination of these cases revealed that only 3 (11%) had a history of previous PTB treatment. Likewise, culture detected 7 (3%) presumptive PTB cases that were missed by Xpert.

**Table 2:** Performance Characteristics of Xpert on sediments and raw sputum samples

Performance characteristics of Xpert in Presumptive PTB using LJ culture as a reference method (*N* = 262)				
Sensitivity (%)	Specificity (%)	PPV (%)	NPV (%)	*P*-value
Performance characteristics of Xpert on sputum sediments using experimental dilution (1:2)				
92 (84–97)	85 (79–90)	74 (67–80)	96 (91–98)	0.0001
Performance characteristics of Xpert on raw sputa using standard dilution (1:2)				
86 (77–93)	86 (77–93)	75 (65–83)	93 (88–96)	0.001
Incremental values				
6	1	1	3	
Performance characteristics of Xpert in HIV Infected presumptive PTB (*n* = 39)				
Performance characteristics of Xpert on sputum sediments using experimental dilution (1:2)				
100 (81–100)	82 (60–95)	81 (64–91)	100	0.0001
Performance characteristics of Xpert on raw sputa using standard dilution (1:2)				
88 (64–99)	86 (65–97)	83 (59–96)	91 (70–99)	0.0001
Incremental values				
12	4	2	9	

NB: The Xpert MTB/RIF assay have been abbreviated as Xpert

## Discussion

The main finding of this study is that sputa sediments to Xpert SR ratio of 1:2 considerably increase the sensitivity of detecting MTB, especially in HIV infected presumptive PTB cases. Our findings are in agreement with those of Dharan et al. [16] who had a high detection rate of MTB when the sediment/SR ratio was lowered to 1:2. These findings support the use of sputum sediments on Xpert especially in a TB and HIV co-infection endemic setting such as Tanzania and across Sub-Saharan Africa. Although this procedure presents additional costs of about 5–10 USD per sample [21] and time for centrifuging the raw sputa, the cost incurred for treating a patient inadequately, especially if cases are missed, outweighs the cost for the modified protocol. Ineffective diagnostics prompts multiple hospital visits causing additional costs for consultation, transportation to health facility and repeated Xpert test in a heavily subsidized health system. For example, in low and middle income countries like Tanzania, the cost of treating a patient with drug susceptible TB ranges from 250-300USD [22].

In the present study, the specificity of Xpert on sputa sediments among HIV infected participants was 4% lower compared to that in raw sputa, as one more non-PTB HIV case was falsely determined as positive on sediments (Table2). Remarkably, the Xpert detected 26% more MTB cases on sputum sediment than LJ culture (Table2). Certainly, the difference could be attributable to inability of Xpert to discriminate between viable and non-viable MTB DNA. This is a common phenomenon in previously treated, PTB cases, resulting in false positives [23, 24]. However, only 11% of the false positive population had a history of previous PTB treatment. This finding, which is in keeping with those by Geleta DA et al. [25], could partly result from participants deliberately giving false TB treatment history [26]. On the other hand, Xpert sediment results had an average of 4% false negatives (Table 2). Indeed, this might be due to a difference in detection limit of Xpert, which requires higher number of AFB than culture methods. For example, the estimated detection limit of Xpert is 131 colonies forming unit (CFU)/ml, while LJ culture is 10–100 CFU/ml [27].

The notable strength of this study is the detection of 7 PTB cases more on the modified protocol for sediments that were missed on raw sputa (Fig. 1). In addition, the Xpert detected 3 out of 262 (1.1%) cases with rifampicin resistance tuberculosis (RR-TB) in both, raw sputa and sediments that were collected from patients. These cases received treatment for multi-drug resistance tuberculosis (MDR-TB) as per existing guideline in Tanzania and elsewhere [28, 29]. This low proportion of RR-TB best align with findings by Nagu et al. who found uncommon cases of MDR-TB among patients without prior exposure to anti-TB drugs in Tanzania [30]. Despite these strengths, our study has some limitations. We were unable to compare the performance characteristics of Xpert with smear microscopy results because KIDH has shifted the practice of using smear microscopy as the frontline TB diagnostic to Xpert as recommended by the WHO. Therefore, we were unable to correlate Xpert performance characteristics with sputum AFB density. However, the CT values have not been shown to correlate with smear for AFB density [31] and hence cannot affect validity of the current findings. Also, our results did not compare the ratio of sputum sediment to Xpert SR of 1:3 to that of modified protocol (1:2) and we recommend further study to decipher any difference in detection of MTB.

## Conclusion

Lowering sediment to SR dilution ratio to 1:2 improved performances of Xpert on Sputum Sediment Samples, especially those obtained from HIV-infected individuals. However, operational and cost-effective studies will be required to determine the feasibility of the national TB program in implementing the proposed modified protocol sustainably.

## Abbreviations

AFB: Acid fast bacilli
AIDS: Acquired Immunodeficiency syndrome
CFU: Colonies forming unit
CLSI: Clinical and Laboratory Standard Institute
CT: Cycle threshold
HIV: Human Immunodeficiency Virus
KIDH: Kibong’oto Infectious Diseases Hospital
LJ: Lowenstein Jensen
MDR-TB: Multidrug resistant Tuberculosis
MTB: *Mycobacterium tuberculosis*
NALC-NaOH: N-Acetyl-L-Cysteine Sodium Hydroxide
NPV: Negative predictive value
PPV: Positive Predictive value
PTB: Pulmonary tuberculosis
RR: Rifampicin resistance
SR: Sample reagent
TB: Tuberculosis
WHO: World Health Organization
Xpert: Xpert MTB/RIF assay
